# Imperfect Isolation: Factors and Filters Shaping Madagascar’s Extant Vertebrate Fauna

**DOI:** 10.1371/journal.pone.0062086

**Published:** 2013-04-23

**Authors:** Karen E. Samonds, Laurie R. Godfrey, Jason R. Ali, Steven M. Goodman, Miguel Vences, Michael R. Sutherland, Mitchell T. Irwin, David W. Krause

**Affiliations:** 1 Department of Biological Sciences, Northern Illinois University, DeKalb, Illinois, United States of America; 2 Department of Anthropology, University of Massachusetts, Amherst, Massachusetts, United States of America; 3 Department of Earth Sciences, University of Hong Kong, Hong Kong, China; 4 Department of Zoology, Field Museum of Natural History, Chicago, Illinois, United States of America; 5 Association Vahatra, Antananarivo, Madagascar; 6 Department of Evolutionary Biology, Zoological Institute, Technical University of Braunschweig, Braunschweig, Germany; 7 Department of Anthropology, Northern Illinois University, DeKalb, Illinois, United States of America; 8 Department of Anatomical Sciences, Stony Brook University, Stony Brook, New York, United States of America; Raymond M. Alf Museum of Paleontology, United States of America

## Abstract

Analyses of phylogenetic topology and estimates of divergence timing have facilitated a reconstruction of Madagascar’s colonization events by vertebrate animals, but that information alone does not reveal the major factors shaping the island’s biogeographic history. Here, we examine profiles of Malagasy vertebrate clades through time within the context of the island’s paleogeographical evolution to determine how particular events influenced the arrival of the island’s extant groups. First we compare vertebrate profiles on Madagascar before and after selected events; then we compare tetrapod profiles on Madagascar to contemporary tetrapod compositions globally. We show that changes from the Mesozoic to the Cenozoic in the proportions of Madagascar’s tetrapod clades (particularly its increase in the representation of birds and mammals) are tied to changes in their relative proportions elsewhere on the globe. Differences in the representation of vertebrate classes from the Mesozoic to the Cenozoic reflect the effects of extinction (i.e., the non-random susceptibility of the different vertebrate clades to purported catastrophic global events 65 million years ago), and new evolutionary opportunities for a subset of vertebrates with the relatively high potential for transoceanic dispersal potential. In comparison, changes in vertebrate class representation during the Cenozoic are minor. Despite the fact that the island’s isolation has resulted in high vertebrate endemism and a unique and taxonomically imbalanced extant vertebrate assemblage (both hailed as testimony to its long isolation), that isolation was never complete. Indeed, Madagascar’s extant tetrapod fauna owes more to colonization during the Cenozoic than to earlier arrivals. Madagascar’s unusual vertebrate assemblage needs to be understood with reference to the basal character of clades originating prior to the K-T extinction, as well as to the differential transoceanic dispersal advantage of other, more recently arriving clades. Thus, the composition of Madagascar’s endemic vertebrate assemblage itself provides evidence of the island's paleogeographic history.

## Introduction

Madagascar has long been recognized as one of the world’s highest biodiversity priorities [1]; its extant vertebrate terrestrial and freshwater vertebrate assemblages are highly distinctive and have high levels of species endemism across groups [2]. However, within some clades, Madagascar’s fauna is taxonomically imbalanced and species-poor. Among mammals, for instance, Madagascar shows conspicuous absences relative to typical African sub-Saharan faunas; if recent introductions by humans are excluded, only seven extant and subfossil placental orders are represented: Bibymalagasia, Afrosoricida, Primates, Chiroptera, Carnivora, Rodentia, and Cetartiodactyla. Similar patterns of disproportionate absences exist in other vertebrate higher taxa (e.g., [3]–[5]). Furthermore, many forms occupy phylogenetic positions that are basal relative to continental members of their respective groups [3], [6], [7].

With regard to direct evidence of the rich vertebrate fauna that once existed, the island is well known for its fossil assemblages (e.g., [8]–[10]). However, the fossil record is largely constrained to three main intervals in Madagascar’s Mesozoic/Cenozoic history – Late Triassic to mid-Jurassic, Late Cretaceous, and Late Pleistocene/Holocene – thus, leaving huge gaps. One gap is particularly critical to the biogeographic origins of the extant fauna - that spanning most of the Cenozoic, which is when many of the extant groups arrived and subsequently evolved (e.g., reviews by [11]–[13]).

On the early side of this gap, a diverse fossil assemblage (including, ray-finned fishes, anurans, turtles, snakes, non-ophidian squamates [‘lizards’], crocodyliforms, birds, and mammals, as well as sauropod and non-avian theropod dinosaurs) from the Late Cretaceous (70–65 million years ago, Ma) is now well-documented from the Maevarano Formation of northwestern Madagascar [10]. With the possible exception of a podocnemidid turtle (cf. Erymnochelys; [14]), the low-level fossil vertebrate taxa described from the Maevarano Formation could not be considered as candidates involved in the ancestry of clades represented in either the subfossil or in the extant fauna of Madagascar [15], [16].

On the recent side of the gap, the earliest Cenozoic vertebrate specimens (with the exception of some mid-Eocene nearshore marine fossils; e.g., [17]) are “subfossils” less than 80,000 years old [18]. Thousands of bones have been described from Late Pleistocene and Holocene deposits including those of crocodylians, turtles, lemurs, bats, carnivorans, rodents, pygmy hippopotamuses, the aardvark-like *Plesiorycteropus*, and various birds, such as the flightless elephant birds (e.g., [9], [19]–[21]). These remains are, for the most part, too recent to contribute direct information to debates concerning the biogeographic origins of Madagascar’s extant vertebrate fauna; their existence generally adds to our knowledge of vertebrate clade origins in Madagascar in the same manner as do extant vertebrates (i.e., through molecular phylogenetics, phylogenomics, biogeographic analysis, etc.). It is by using such analytical tools that some vertebrate groups (e.g., microhylid frogs, oplurid lizards, boid and xenotyphlopid snakes) that are not represented in the Mesozoic fossil record of Madagascar are nevertheless inferred to have arrived (or originated) there prior to the K-T extinctions, but not without contention, while most others (e.g., other frogs, snakes, lizards, many birds, and all mammals) are thought to have arrived after this event, when Madagascar was fully isolated. It is this type of evidence that overwhelmingly supports a greater role for dispersal than vicariance in explaining the island’s extant vertebrate fauna. Current evidence lends little support for the vicariance scenario for most of the island’s extant vertebrate clades [13], [22].

These inferences, though important, focus only on the two endpoints – clade origins and their modern or subfossil descendants. What is missing from our current understanding is an examination of temporal change in Madagascar’s vertebrate fauna – specifically how changes in clade representation correlate with particular paleogeographical events, and how the history of changes in vertebrate clade representation, by itself, informs our understanding of the island’s global isolation.

This is the primary goal of this paper. We examine Madagascar’s changing vertebrate clade profiles (percentages of fishes, amphibians, reptiles, birds, and mammals) through time within the context of its geologic history, to evaluate the extent of the island’s biogeographic isolation and the principal factors that have molded the composition of its extant (and subfossil) vertebrate fauna. We conclude that two events in particular, the end-Cretaceous extinctions and the shift in prevailing ocean current flow in the mid-Miocene, help to explain observed changes in the representation of the major groups of vertebrates (and in the characteristics of that vertebrate fauna). We also infer that the composition of Madagascar’s extant and subfossil vertebrate fauna owes more to events that occurred after the K-T extinction than to its history of colonization and evolution during the Mesozoic.

## Materials and Methods

Data reflecting our current understanding of the biogeographic history of the extant Malagasy vertebrate fauna and key geological, geophysical, and paleoceanographic information were compiled to address impacts on Madagascar’s vertebrate fauna by two key events – the mass extinction at the Cretaceous-Tertiary (K-T) boundary and the shift in prevailing ocean flow during the Miocene [23].

For each Malagasy vertebrate “clade” (defined as a group of Malagasy species descended from a single ancestral species that arrived on Madagascar either via dispersal or vicariance), we recorded class, time of arrival, ancestor type (obligate freshwater, terrestrial, facultative swimmer, volant), overwater dispersal ability, geographic source area, occurrence in the Malagasy fossil record (Mesozoic or Quaternary), and whether there are representative extant members (Table S1). With a few exceptions, dispersal ability was coded as either dispersal-disadvantaged (obligate freshwater or terrestrial), or dispersal-advantaged (facultative swimmer or volant). Vertebrate profiles were then compared before and after specific events. Estimates of arrival time and source area were drawn from either published clock dates, or from molecular phylogenies combined with cladograms based on morphological data (some including fossil taxa). The prevailing direction of ocean current at the time of colonization was only scored for clades arriving during the Cenozoic owing to the limited availability of paleocurrent modeling for earlier times. We used SPSS (version 20) and Stata SE (version 11) to explore changes in class, geographic source, dispersal advantage, locomotor type, and percentage extinct, from before to after the selected events. In addition to providing standard Pearson’s chi-square values for each row-by-column frequency table, we present Fisher’s exact test results. The latter is preferable to traditional Pearson’s chi-square or the maximum likelihood test for independence when one or more anticipated values are small, particularly when expected values are less than five in one or more of the cells, or when marginal values are strikingly uneven. Fortunately, Fisher’s exact test is a combinatorially exhaustive calculation that provides the exact probability of finding any given result (or more extreme difference) by chance alone [24]. As it is an exact test, it delivers a probability value without degrees of freedom. We also used SPSS’s loglinear function to elucidate the simultaneous effects of geographic source, vertebrate class, and time.

Tetrapod representation (percentages of amphibians, reptiles, birds, and mammals) was also compiled globally (based on counts of families recorded by [25], [26]) and within Madagascar (based on counts of inferred independent arrivals) for three periods (the Late Cretaceous, Paleogene, and Neogene). For Madagascar, some analyses involve “profiles” (the inferred fauna present at a given time interval, while others use “arrivals” (the group of clades arriving within that time interval). Our goal here was to evaluate the degree to which Madagascar was insulated from the rest of the world by addressing a series of questions. Is Madagascar’s tetrapod diversity today (or during the very recent past) more a by-product of its dispersal history during the Cenozoic or of its earlier vicariance history? In other words, is the character of Madagascar’s tetrapod fauna ancient? Do the tetrapod clades arriving during the Cenozoic match (in percentage class representation) Cenozoic clades outside of Madagascar? To evaluate similarities and differences over time, we calculated assemblage proximities based on vector correlations and produced a dendrogram showing the average linkages of seven vertebrate assemblages –Madagascar in the Late Cretaceous, Paleogene, Neogene and Quaternary, and the globe during the Late Cretaceous, Paleogene and Neogene (Sahney’s tetrapod familial counts at 70, 35, and 10 million years ago) [25].

## Results

### Before and at the Cretaceous-Tertiary Boundary

#### Rifting of madagascar from africa

**Figure 1:**
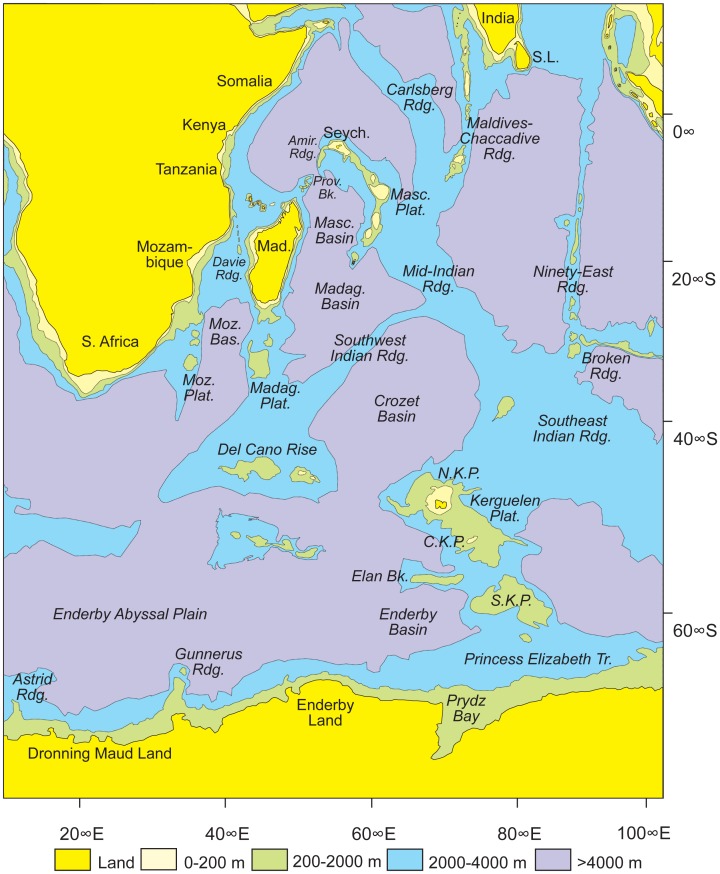
Simplified bathymetric map of the western and central Indian Ocean, and adjacent Southern Ocean. Note that the 0–200 m bathymetric interval around Antarctica is not portrayed due to a lack of detailed information in the areas adjacent to the continent. Based on the GEBCO [153] chart.

At approximately 165 Ma, the southern supercontinent of Gondwana split into a western block composed of Africa and South America, and an eastern entity made up of Madagascar, Seychelles, the Indian subcontinent, Antarctica, and Australia [27], [28]. During this spreading phase, which lasted until about 116 Ma [29], the approximately N-S aligned Davie Ridge Fracture Zone, which bisects the Mozambique Channel, operated as a dextral (far-side-moves-to-the-right) transform fault [30]. Consequently, ocean floor was generated and spread in the western Somali Basin (Fig. 1), east of modern-day Kenya-Tanzania, as well as in the Mozambique and Enderby Basins (between southern Madagascar and Antarctica). It is worth noting that, for most of this time, eastern Africa and western Madagascar would have been close, as East Gondwana migrated slowly southwards relative to its western counterpart. Only after the M11 magnetic period ( = magnetochron) (∼136 Ma [31]), did the gap between the two landmasses grow more rapidly ([28]; see their Fig. 13).

#### Isolation of indo-Madagascar

The isolation of Indo-Madagascar (a landmass including Madagascar, Seychelles, and the Indian subcontinent that existed for some 30 million years) from its former Gondwanan neighbors was largely completed by ∼115 Ma as the southeastern part of the subcontinent peeled away from the Gunnerus Ridge-Enderby Land margin of eastern Antarctica [32]. Perhaps of most relevance for this study is the fact that Madagascar has been in the same position relative to mainland Africa ever since, separated by a minimum distance of 400 km.

#### Kerguelen plateau

For some time, based on the paleogeographic reconstructions and inferences of Hay *et al*. [33], it has been proposed that South America and Indo-Madagascar were indirectly linked via Antarctica and the now largely submerged Kerguelen Plateau (Fig. 1), thereby potentially explaining the close relationships of various latest Cretaceous taxa on the two disjunct land masses (e.g., [15], [16], [34]–[36]). Recently, the Kerguelen Plateau land-bridge hypothesis was explored in two publications [32], [37]. Based on available geological data, and drawing upon geophysical modeling, the 2011 study included four detailed paleogeographical reconstructions: mid-Early Cretaceous, 120.4 Ma; Early/Late Cretaceous boundary, 99.6 Ma; mid-Late Cretaceous, 83.5 Ma; and end-Late Cretaceous, 67.7 Ma. The critical findings were that large portions of the Kerguelen Plateau may have been emergent up until the Early/Late Cretaceous boundary but that Indo-Madagascar was still separated from the closest emergent parts of this plateau by some 1,900 km. Furthermore, by 95–90 Ma, only small portions of the terrain could have been above sea level in a vast and still expanding southern Indian Ocean. Another critical aspect is that the plateau never directly abutted Antarctica – the highs on the edifice being separated from the continent by deep waters over the Princess Elizabeth Trough, a barrier that would have been >300 km wide. Therefore, any terrestrial animals passing between Antarctica and Indo-Madagascar during the middle and Late Cretaceous could not have passed via a land-bridge; they would have needed to make substantial overwater journeys. Correspondingly, Ali & Krause [32] demonstrated with stratigraphically calibrated phylogenies that those latest Cretaceous Malagasy vertebrates judged to be obligatorily terrestrial and poor overwater dispersers (e.g., large dinosaurs, notosuchian crocodyliforms) had long ghost lineages, extending into the Early Cretaceous, thereby indicating that their ancestors had likely arrived on Indo-Madagascar before it was isolated from the rest of Gondwana.

#### Separation of the indian subcontinent from madagascar

Separation of the Indian subcontinent and Madagascar occurred approximately 88 Ma [38], [39]. During the final part of the Cretaceous, the Indian subcontinent and Madagascar might have been indirectly “connected” by land on Providence Bank and Amirante Ridge [as well as the Seychelles Block; 27], but such a route was likely broken by expanses of ocean several tens to a few hundred km in width.

#### K-T boundary extinction

A catastrophic, global, mass extinction occurred at the boundary between the Cretaceous and Tertiary, with >75% of the world’s species going extinct [40]. The event is widely attributed to an asteroid impact in Mexico (e.g., [41]), though other associated global effects such as massive volcanic eruptions, acid rain, atmospheric dust, temperature and sea level changes also likely played a critical role in these extinctions [42].

The formation of the Deccan traps in northwestern India between 60 and 68 million years ago (eruptions that may have lasted less than 30,000 years total) also likely contributed to the extinctions at the Cretaceous-Tertiary boundary, releasing sulfur dioxide and other volcanic gasses into the atmosphere [43]. The proximity of the Deccan traps to Madagascar, and the associated ash falls and depleted photosynthesis likely played a major role in the extinction of the island’s groups.

#### Relevant biogeographic events

Various archaic groups of actinopterygian fishes inhabited Madagascar's freshwaters prior to the Cenozoic, comprising at least three families that have a Cretaceous fossil record but are now locally extinct (Lepisosteidae, Phyllodontidae, and Pycnodontidae). The origins in Madagascar of five families of more derived actinopterygians represented in the extant fauna (Aplocheilidae, Bedotiidae, Cichlidae, Clupeidae, and Mugilidae) are usually also reconstructed in this time period [44]–[48]. An ancient Gondwanan origin has also been reconstructed for an enigmatic group of cave-dwelling fish (i.e., Milyeringidae) that very recently have been demonstrated to represent a compelling example of Australian-Malagasy sister relationships [49], [50]. For the extant fish groups, our analysis is based on the prevailing assumption of ancient vicariance; one recent study indicates a possible younger origin of these fishes (see below) [51]. Two amphibian groups may also have ancient biogeographic links: a Late Cretaceous giant ceratophryine frog with South American affinities that has no living representatives on Madagascar [52], and some of the island’s microhylid frogs (the clade composed of Cophylinae and Scaphiophryninae, and the endemic Mantellidae; [51], [53]).

Malagasy podocnemidid turtles are attributed to Mesozoic vicariance, as they share close phylogenetic affinities to turtles from South America [36]. A lower jaw of cf. *Eremnochelys* from the Late Cretaceous of Madagascar [14] supports this scenario; these fossils represent the only Cretaceous occurrence of a genus currently represented in the extant Malagasy vertebrate fauna [10]. The bothremydid turtle *Kinkonychelys* is also described from the Late Cretaceous [54], as well as *Sokatra*, a more basal pelomedusoid [55]. *Kinkonychelys* is interesting biogeographically in that it is nested within a tribe (Kurmademydini) that is otherwise only known from India, thus reflecting the relatively recent physical connections between Madagascar and the Indian subcontinent (at 85–90 Ma).

The first definitive record of Mesozoic lizards from Madagascar is a questionably-identified cordylid lizard from the Late Cretaceous [10]; representatives of the Cordylidae are today restricted to sub-Saharan Africa. Other Late Cretaceous reptiles include at least four species of notosuchian and two species of neosuchian crocodyliforms [56]–[58], three species of basal snakes [59], two species of sauropod dinosaurs [60], and three species of non-avian theropod dinosaurs [61]–[63]; none of these taxa appears to be relevant to the ancestry of extant Malagasy clades. Based on molecular evidence, other extant Malagasy reptiles whose ancestors are thought to have arrived before the K-T boundary include oplurid lizards and boid snakes [36], and the endemic family of Malagasy blind snakes Xenotyphlopidae [64]. Additionally, zonasaurine gerrhosaurid lizards are thought to have arrived through a single dispersal event from Africa approximately 66 Ma [65].

The ancestral stocks of all of the island’s flightless or flight-limited birds (e.g., Aepyornithidae, Mesitornithidae) are thought to have inhabited the island during the Mesozoic, as they either have phylogenetic affinities to taxa on southern continents or Asia, or belong to lineages with inferred minimal geological vicariance ages of 100–80 Ma [66] (but see discussion in [67] suggesting a Miocene arrival for some taxa). To date, however, the Late Cretaceous fossil record provides little support for vicariance; it has yielded only a very primitive avifauna, comprised of at least six basal (non-neornithine) taxa [68], [69].

The Mesozoic record of mammals from Madagascar is limited to a Jurassic australosphenidan [70] and several Late Cretaceous non-placental taxa, none of which played a role in the ancestry of the extant clades of placental mammals that live on the island today [16], [71]–[73].

For several extant clades of Malagasy vertebrates assumed to have arisen through Mesozoic vicariance, the relationships or divergence ages are contentious. While some molecular timetrees have reconstructed the ages of the three main freshwater percomorph families (cichlids, bedotiids, aplocheiloids) as Mesozoic (e.g., [74]), others have favored their origin in the Paleogene [51], [75]. A recent molecular analysis of teleost fishes (based on a large number of nuclear genes) [76] obtains relatively young Cenozoic ages for the percomorph radiation that are at odds with the orthodox view of an ancient vicariant origin of these animals in Madagascar. Near et al. [76] also provide compelling evidence of prior overestimation of divergence dates by mtDNA analyses (such as those of [74]). On the other hand, for fishes like the Malagasy cave-dwelling freshwater eleotrids (Typhleotris, an endemic genus sister to the Australian Milyeringa), a long-distance marine dispersal is hard to imagine [49]. A more in-depth analysis of this question is necessary. Similarly, affinities of the giant Cretaceous frog Beelzebufo to South American ceratophryids are supported by osteological characters [52] but are in disagreement with hypotheses of younger origins of this Neotropical family [77]. Malagasy boid and xenotyphlopid snakes might be slightly younger as well and could have arrived in Madagascar around the K-T boundary or shortly thereafter [51].

### After the Cretaceous-Tertiary Boundary Until the Mid-Miocene

#### Paleocene/Eocene thermal maximum

The beginning of the Eocene (55.8 Ma) was marked by a dramatic global climatic perturbation [78], which lasted around 200,000 years [79]. The change appears to have been rapid; within a few thousand years, sea-surface temperatures increased by 5–9°C [78], [80]–[83]. The temperature shift coincided with rapid range changes for terrestrial plants and the elimination of large numbers of benthic foraminiferan species, while other taxa such as planktonic foraminifers, dinoflagellates, and mammals flourished.

#### Grand coupure/Mongolian remodeling

The Eocene/Oligocene boundary was marked by an extreme lowering of temperature and sea level in Europe and Asia, concurrent with the beginning of the formation of the Antarctic ice cap [84], [85]. This change in climate coincided with large-scale extinctions and faunal turnover.

#### Establishment of the current monsoon pattern

The monsoon system that currently affects South, Southeast, and East Asia probably initiated at or near the start of the Miocene. This is supported by the coincident onset of desertification in Asia [86] and a marked influx of clastic sediments to Asia’s marginal basins and oceans [87]. Furthermore, Cenozoic deposits in China reveal a marked shift in plant cover at the Oligocene-Miocene boundary [88]. By 15 Ma, the modern oceanic circulation pattern was likely well established.

#### Beginning of ocean current shift

The tipping point for the change to the “modern” circulation in the Southwest Indian Ocean [89] probably occurred during the mid-Miocene when Madagascar’s northern extremity (now at 12°S) began to impinge upon the South Equatorial Current, which flows E to W across the Indian Ocean basin, its southern edge presently being positioned at ∼17°S [90], [91]. Plate modeling suggests that this occurred ∼20 Ma [23]. Prior to this, and from at least the start of the Cenozoic [23], the W to E flow around Madagascar at the height of each austral summer was 13–23 cm/s between NE Mozambique-SE Tanzania and Madagascar. Such speeds would have enabled mammals adrift on rafts (e.g., vegetation mats, trees) off eastern Africa the potential to cross the Mozambique Channel in as few as 30 days.

#### Relevant biogeographic events

There is compelling evidence for dispersal of hyperoliid frogs from Africa to Madagascar at 19–30 Ma (Heterixalus) and from Madagascar to the Seychelles at 11–21 Ma (Tachycnemis). These genera are highly nested among African genera and a molecular clock estimate places their divergence within the Cenozoic [92], [93]; slightly older dates for their origin [51] might be due to the use of different African representatives in the timetree. The endemic subfamily Dyscophinae is thought to have dispersed to Madagascar via India between 39–76 Ma [53], [94]. Closest relatives of the Mantellidae are mainly Asian; their phylogenetically nested position suggests dispersal from India, probably during the Early Paleocene [53], [95].

Chameleons are hypothesized to have originated on Madagascar [7], and to have experienced subsequent and repeated Cenozoic dispersals to continental Africa and to Comoros [7], [51]. Recent work has demonstrated a further dispersal from Africa to the Seychelles 34–38 Ma [96], [97] and postulated origins in Africa rather than Madagascar [97]. The Malagasy gecko *Blaesodactylus* is thought to have arrived during the Paleocene-Eocene [98]. Most skinks arrived through dispersal from Africa [99], while one (e.g., *Cryptoblepharus*) appears to be the result of a single overwater dispersal from Australia or Indonesia, with subsequent colonization of continental Africa, Mauritius, and the Comoros [100].

Evidence suggests that most lamprophiid snakes share affinities with African taxa, and reached Madagascar <31 Ma [101]. Typhlophid snakes are also thought to have arrived after the K-T boundary, as *Typhlops* has a molecular divergence time 59–63 Ma [64].

Bird groups arriving after the Cretaceous, but before the mid-Miocene, include the families Bernieridae, Campephagidae, Eurylaimidae, Psittacidae, and Vangidae [102]–[106]. These events are reconstructed as dispersals from multiple geographic areas and, in some cases, multiple dispersals within families [107].

Molecular work provides strong evidence that Madagascar’s four extant non-volant native mammal groups – lemuroids, carnivorans, tenrecs, and nesomyine rodents – arrived between the end-Cretaceous and the mid-Miocene, probably all from Africa. Lemurs arrived first (50–54 Ma), followed by tenrecs (25–42 Ma), carnivorans (19–26 Ma), and then nesomyine rodents (20–24 Ma) [108]–[112]. African affinities currently seem to be clear for all four clades, but have been debated for lemuroids and carnivorans [113]; but see critique by [114].

The Malagasy Holocene subfossil record contains a bizarre mammal with unclear affinities: *Plesiorycteropus*. This highly distinct and unusual genus is placed in its own order (Bibymalagasia [19]). Historically, the mammalian groups with which it was thought to be allied are all ancient, suggesting a very old origin, likely through vicariance. However, some analyses [115], [116] suggest a sister taxon relationship with extant aardvarks (*Orycteropus*), nested within the Afrotheria [117], which would support a more recent African origin.

Three families of bats (Emballonuridae, Myzopodidae, and Nycteridae) have molecular divergence times within the Paleogene [118]. However, because these times are all maxima, it is difficult to determine when the colonizations occurred, as actual arrival dates may have been more recent.

### After the Mid-Miocene

#### Shift in ocean currents in the SW indian ocean

**Figure 2:**
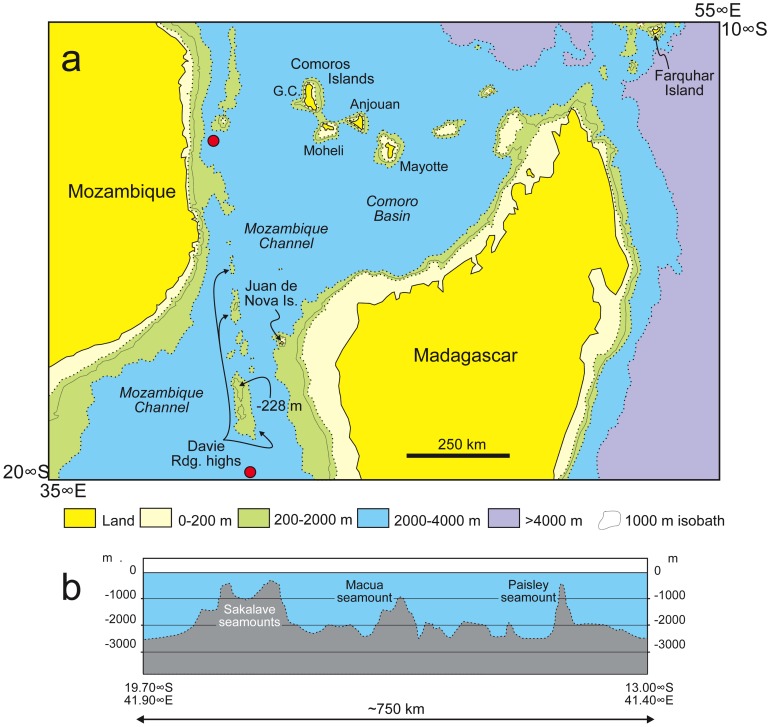
Simplified bathymetric map of the Mozambique Channel area. The relative positions of Madagascar, Mozambique, and nearby islands are shown in (a), while (b) shows a bathymetric cross-section along the Davie Ridge. Red dots on (a) indicate the two ends of the cross-section. Even if elements of the ridge were subaerial, the deep, broad troughs widely separating the peaks would have posed formidable barriers to obligatorily terrestrial animals.

Paleoceanographic modeling indicates that the present-day E to W surface-water direction in the SW Indian Ocean, which is not conducive to Africa-to-Madagascar migrations, was in operation by at least 15 Ma (Fig. 2; [89]). This appears to have brought about a major change in the ability of obligate rafters (dispersal-disadvantaged animals) to colonize Madagascar [13], [23].

#### Intensity of the monsoon system

The monsoon system has not remained stable and data from drill cores from the Arabian Sea indicate that it became more intense during the Late Miocene [119], [120].

#### Relevant biogeographic events

The amphibian Ptychadena mascareniensis is thought to have arrived on Madagascar through overwater dispersal from Africa during the Plio-Pleistocene and subsequently introduced by humans to the Mascarene and Seychelles islands [121], [122]. Both extant and subfossil tortoises are thought to have arrived during this time [123]. Palkovacs et al. [124] suggest that Madagascar was colonized by tortoises once, 17.5–11.5 Ma, and from which species subsequently dispersed to other western Indian Ocean islands.

The extant Nile crocodile (*Crocodylus niloticus*) and the subfossil *Voay robustus* [125] are not closely related to the island’s Late Cretaceous crocodyliforms [56]; evidence suggests that both recent forms arrived through dispersal from Africa after the mid-Miocene, likely through two independent events. *Voay* is closely related to the African dwarf horned crocodile (*Osteolaemus*), while the Malagasy *Crocodylus niloticus* cannot be specifically distinguished from its counterpart in mainland Africa [125]–[128].

The house geckos Hemidactylus mercatorius and H. playcephalus, once believed to have been translocated to Madagascar by humans, are now thought to have arrived through two separate events prior to human colonization of the island [98]. One genus of lamprophiid snakes (Mimophis) is also thought to have colonized Madagascar during this time (<13 Ma [101]). Most bird groups also arrived after the mid-Miocene, including the families Dicruridae, Motacillidae, Nectarinidae, Pycnonotidae, and certain Strigidae, Sturnidae, and Zosteropidae [107], [129]–[133].

Hippopotamuses are believed to have arrived in the latest Cenozoic, but not via human agency [21], [134] and, according to historical accounts, persisted after humans arrived [135]. Despite having been common in Madagascar during the Holocene, the three endemic Malagasy hippopotamus species are now extinct. Hippopotamus laloumena and H. lemerlei are related to the Nile hippopotamus (H. amphibius; [134]), while Hexaprotodon guldbergi [136] is more closely related to the Liberian pygmy hippopotamus (He. liberiensis; [137]); hence, a minimum of two overwater crossings from Africa is implied.

The only other mammals to arrive during this period are bats. Most families are thought to have colonized Madagascar during the last 15 Ma, many probably during the last 5 Ma [138]. Colonization events include three within Hipposideridae, at least eight within Molossidae, three within Pteropodidae, and four within Vespertilionidae [118], [139]–[142]. Bats therefore show a different pattern from non-volant endemic mammals; they contain species nested within distantly-related families, some with cosmopolitan distributions [118], having arrived through multiple dispersal events from at least two source continents.

Lastly, a number of wild mammal species (e.g., shrews, wild pigs, rats) are thought to have been introduced to the island by early human settlers (Table 1; [143]–[145]; these taxa were excluded from our analysis. Details of their arrivals are unknown, and it is possible that some of them arrived via overwater dispersal prior to human colonization.

**Table 1:** Taxa probably introduced by early human settlers in the Holocene (last 2,000 years).

Family	Scientific Name	Type	Time	Notes	References
Ranidae	*Hoplobatrachus*	Terrestrial semi-aquatic	Holocene	Source populations from India	[154]
Pelomedusidae	*Pelomedusa*	Terrestrial semi-aquatic	Holocene	Source populations probably from eastern Africa	[155]
Gekkonidae	*Hemidactylus frenatus*	Terrestrial	Holocene	Asia	[98]
Muridae	*Rattus rattus*	Terrestrial	Holocene	Source populations from India	[156]
Muridae	*Mus musculus*	Terrestrial	Holocene	Source population from Yemen	[157]
Soricidae	*Suncus murinus*	Terrestrial	Holocene	Source populations from Asia, probably introduced along Arabian marine traffic routes, as was *Mus musculus*	[143]
Soricidae	*Suncus etruscus* (ex. *madagascariensis*)	Terrestrial	Holocene	Possible human introduction from Asia	[145]
Suidae	*Potamochoerus larvatus*	Terrestrial	Holocene?	Thought to have originated by human introduction, but molecular data missing	[144]

### Profile Summary and Comparative Synthesis

**Figure 3:**
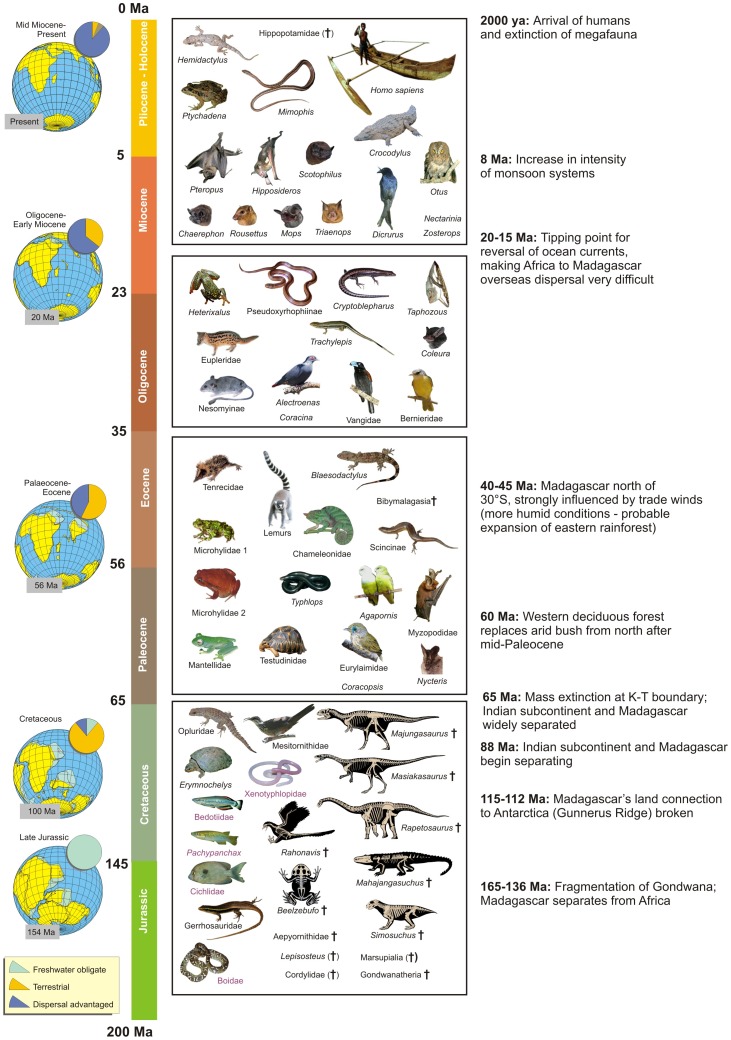
Timetable summarizing major paleogeographical and paleoclimatic events relevant to the biogeographic history of Madagascar. Table includes a non-exhaustive summary of vertebrate taxa that arrived or were present on the island during four major intervals (as in [13]; herein, taxa from the second and third period were merged for analysis). The box for the Jurassic-Cretaceous shows extinct fossil taxa that were present in Madagascar in the Late Cretaceous, plus extant taxa reconstructed to have existed during this time. Subsequent boxes show taxa that are estimated to have arrived on the island during the respective interval, with each image representing one (or rarely two) endemic Malagasy clades. Extinct taxa are marked with † (in parentheses if taxon is extinct on Madagascar but surviving elsewhere). Red font marks taxa that might be of younger origin according to some molecular estimates. Maps show changing landmass configurations and patterns of vertebrate appearance in or colonization of Madagascar by time slice and proportion of dominant colonizer types (unadjusted frequencies in percent, after [13]).

Of the 99 vertebrate “arrivals” to Madagascar tabulated here, some have left no Holocene descendants; however, at least 73 of these have given rise to members of Madagascar’s extant vertebrate fauna and an additional five clades (Aepyornithidae, *Hippopotamus*, *Hexaprotodon*, *Plesiorycteropus*, and *Voay*) that survived into the Quaternary but are now extinct (Fig. 3). The 73 extant vertebrate clades include six clades of bony fishes, five amphibians, 17 reptiles, 18 birds, and 27 mammals (Table S1). In Table 2 we present the dominant clade profile changes across the K-T boundary and in Table 3 the same for the Cenozoic (especially before and after the direction of dominant ocean currents shifted during the mid-Miocene).

**Table 2:** Comparison of vertebrates arriving before and after the K-T boundary.

Question, Null Hypothesis, and Interpretation	Pearson’s Chi SquareValue, df, P	Fisher’s ExactTest P
*Does vertebrate class representation of arrivals change from before to after the K-T boundary?*		
H_0_: There is no change in vertebrate class representation of clade ancestors arriving before vs. afterthe K-T boundary. Reject. Conclusion: Following the K-T boundary, there was an increase inthe percentages of mammalian and avian clade arrivals.	34.999, df = 4, P<0.001	<0.001
*Are these changes reflected in the Late Quaternary and extant fauna profiles?*		
H_0_: There is no difference in the age of clades represented in the Late Quaternary or extant faunasby vertebrate class. Reject. Conclusion: The younger clades represented in the Late Quaternaryand extant faunas of Madagascar are primarily birds and mammals.	34.159, df = 4, P<0.001	<0.001
*Does clade age affect extinction?*		
H_0_: There is no difference in the percentages of vertebrate clades arriving before or after theK-T boundary that have become extinct. Reject. Conclusion: Clades arriving during the Mesozoicare far more likely to be extinct today than those arriving during the Cenozoic.	35.695, df = 1, P<0.001	<0.001
*Among vertebrates present during the Mesozoic, does likelihood of extinction vary by class?*		
H_01_: There is no difference in survival of Mesozoic fishes vs. tetrapod clades. Reject. Conclusion:Fishes are more likely than tetrapods to have survived.	4.523, df = 1, P = 0.03	0.05
H_02_: There is no difference in survival of Mesozoic tetrapod clades by class. Fail to reject.	1.716, df = 3, P = 0.633	0.8

**Table 3:** Comparisons of vertebrates arriving during the Cenozoic.

Question, Null Hypothesis, and Interpretation	Pearson’s Chi squareValue, df, P	Fisher’s ExactTest P
*Is there a difference in vertebrate class representation of tetrapod arrivals from Africa vs. Asia* *(the Indian subcontinent and Southeast Asia)?*		
H_0_: There is no difference in tetrapod class representation between those arriving from Africavs. Asia during the Cenozoic. Reject. Conclusion: Mammals and reptiles are far more likely thanbirds to have arrived from Africa than from Asia.	19.293, df = 3, P<0.001	<0.001
*Is this source difference dependent on the direction of ocean currents?*		
H_01_: There is no difference in tetrapod class representation of African vs. Asian arrivals beforethe mid-Miocene. Reject. Conclusion: Before the shift in ocean currents, mammals andreptiles are more likely than birds to have arrived from Africa.	8.603, df = 3, P = 0.035	= 0.017
H_02_: There is no difference in tetrapod class representation of African vs. Asian arrivals afterthe mid-Miocene. Reject. Conclusion: After the shift in ocean currents, mammals andreptiles are more likely than birds to have arrived from Africa.	13.03, df = 3, P = 0.005	= 0.005
H_03_: There is no change in overall tetrapod class representation among arrivals before andafter the mid-Miocene. Fail to reject. Conclusion: Both before and after the mid-Miocene,birds and mammals dominate arrivals.	2.653, df = 3, P = 0.448	= 0.482
H_04_: There is no change in geographic source among arrivals before and after the mid-Miocene.Fail to reject. Conclusion: Both before and after the mid-Miocene, most colonizersarrive from Africa.	3.097, df = 3, P = 0.377	= 0.348
*What, then, was the effect of the shift in ocean currents?*		
H_01_: There is no change in dispersal advantage of vertebrate arrivals from before to afterthe mid-Miocene. Reject. Conclusion: After the mid-Miocene, the percentage ofdispersal-disadvantaged taxa colonizing Madagascar decreases.	17.392, df = 1, P<0.001	<0.001
H_02_: There is no change in locomotor type of vertebrate arrivals from before to after themid-Miocene. Reject. Conclusion: After the mid-Miocene, the percentage of volantcolonizers increases markedly.	15.335, df = 2, P<0.001	<0.001

#### Across the K-T boundary

Vertebrate class representation among arrivals changes significantly from before to after the K-T boundary (Table 2). Prior to this period, reptiles represent 51.4% of the arriving vertebrate clades, followed by fishes (25.7%), birds (8.6%), mammals (8.6%), and amphibians (5.7%). After the K-T boundary, mammals (44.3%) and birds (27.9%) dominate among arrivals, followed by reptiles (21.3%) and, finally, amphibians (6.6%).

The extant vertebrate fauna reflects these shifts –78.6% of the Quaternary (including extant) clades that are apparently derived from the Mesozoic Malagasy fauna are reptiles or fishes, while 72.1% of those that derive from Cenozoic arrivals are mammals or birds. Of the 75 Quaternary vertebrate clades scored (an additional 3 were not included, as they could not be reliably scored for arrival time), 100% (27/27) of the mammalian clades, 89.5% (17/19) of the bird clades, 80% (4/5) of the amphibian clades, and 72.2% (13/18) of the reptilian clades derive from Cenozoic colonizers, while none of the fishes (0/6) do (Table 2).

Of the 35 vertebrate lineages known or hypothesized to have arrived during the Mesozoic, 22 (62.9%) are now extinct (although one of these, Aepyornithidae, was still extant in the Late Quaternary) and 13 (37.1%) are still extant. Of the 61 vertebrate clades hypothesized to have arrived during the Cenozoic, only four (6.6%) are now extinct. This difference is highly significant statistically (Table 2). The 22 extinct clades known only from the Mesozoic fossil record may have succumbed to extinction at the K-T boundary, though the virtual absence of Cenozoic terrestrial fossils largely precludes direct testing of this hypothesis.

For vertebrate clades arriving in the Mesozoic, the likelihood of clade extinction varies by class. Fishes are most likely to have survived (66.7%, or 6/9 clades), while tetrapods show much higher losses, with only 26.9% (7/26 clades) surviving to the present. Within tetrapods, however, there is no class-related extinction bias. The relative percentages of amphibian, reptilian, avian and mammalian clades do not differ significantly between survivors and those that became extinct (Table 2). Reptiles dominate both (71.4% of the surviving Mesozoic tetrapod clades and 68.4% of the extinct Mesozoic tetrapod clades).

#### Within the cenozoic

The tetrapod arrivals from Africa (72.9% of all colonizers) and Asia (Indian subcontinent and Southeast Asia combined; 23.7% of all colonizers) during the Cenozoic differed in vertebrate class composition (Table 3). Vertebrate colonizers arriving from Africa are predominantly mammals (55.8%) and reptiles (23.3%), while vertebrate colonizers arriving from Asia are predominantly birds (71.4%). Amphibians represent only 7.0% of Cenozoic tetrapod colonizers, with 50% arriving from Africa and 50% from Asia. Almost all of Madagascar’s Cenozoic mammalian colonizers (96% or 24/25) arrived from continental Africa. The same applies to reptiles (90.9% or 10/11), but not to birds. Only 41.2% of avian colonizers (7/17) arrived from Africa.

This source difference in class profile appears to have been unaffected by the shift in direction of the ocean currents that occurred during the mid-Miocene. Both before and after the shift in ocean currents, mammals and reptiles were more likely than birds to have arrived from Africa. Furthermore, both before and after the mid-Miocene, between two-thirds and three- quarters of colonizers arrive from Africa, and one-quarter to one-fifth from Asia (Table 3). In other words, there is a significant source-by-class interaction, but there is no significant source-by-class-by-time interaction, as demonstrated using a hierarchical loglinear model.

There are no significant changes in the class composition of arriving tetrapods across the mid-Miocene (Table 3). Changes in the relative percentages of vertebrate colonizers belonging to different classes are small. Prior to the mid-Miocene, the percentages of bird (25.9%) and reptile (25.9%) colonizers are identical; mammals (37.0%) surpass both, and all three, in turn, each well surpass amphibians (11.1%). After the mid-Miocene, the dominance of mammalian colonizers increases (50.0%), and avian colonizers (29.4%) are significantly more common than reptilian (17.6%) and amphibian (2.9%) colonizers. However, the overall pattern is the same, with mammals arriving in the highest numbers, followed by birds and reptiles, and then amphibians.

There are no significant changes in the geographic source of the colonizers from before to after the mid-Miocene. During the Cenozoic but prior to the shift in ocean currents, 65.4% of vertebrate colonizers arrived from Africa, while 26.9% arrived from the Indian subcontinent or Southeast Asia, and trivial percentages from elsewhere. After the mid-Miocene, 78.8% of colonizers arrived from Africa, and 21.2% arrived from the Indian subcontinent or Southeast Asia. Despite poor conditions for west-to-east overwater dispersal after the mid-Miocene, colonizers continued to arrive in high numbers. Indeed, during the 15 million years after the mid-Miocene, 34 colonizers arrived; this contrasts with the inferred 27 colonizers that arrived over 50.5 million years of the Cenozoic prior to the mid-Miocene (see reference [13] for discussion of the implications of this patterns for clade extinction, and ghost lineages).

What, then, was the effect of the shift in ocean currents? The important changes from before to after the mid-Miocene are a reduction in the percentage of dispersal-disadvantaged taxa colonizing Madagascar, and a substantial increase in the percentage of volant taxa (Table 3). Dispersal-advantaged taxa move from having had a small advantage (51.9%) to a large advantage (97.1%). Thus, whereas class and source representation remains almost constant, the type of bird and mammal colonizers changes. After the mid-Miocene, most of the mammals that arrived were bats or facultative swimmers instead of terrestrial rafters.

On average, over the entire Cenozoic, volant colonizers outnumber terrestrial ones by a ratio of approximately 2∶1, and they outnumber facultative swimming colonizers by a ratio of over 7∶1. However, these ratios are not constant throughout the Cenozoic. Prior to the mid-Miocene, the ratio of volant to terrestrial colonizers is approximately 1∶1, whereas after the mid-Miocene the ratio is 6∶1. All facultative swimmers appear to have arrived during the latter period, when, like terrestrial rafters, they are outnumbered by volant colonizers (5∶1). In all, most (78.9%) of the known terrestrial colonizers arrived when currents favored west-to-east dispersal from Africa (prior to the mid-Miocene), while only 32.4% of the known volant colonizers arrived during this period.

**Figure 4:**
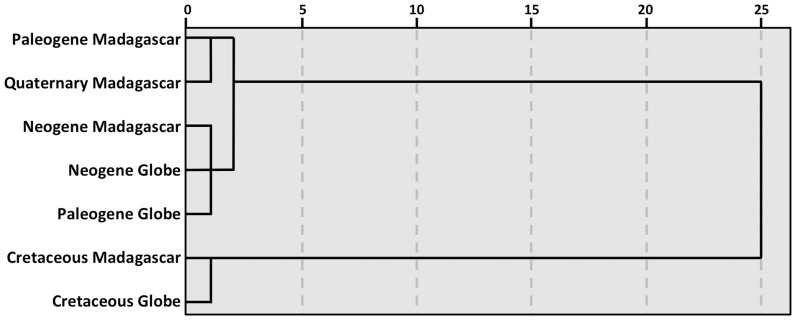
Dendrogram based on the correlations between assemblages or the averages of assemblages, joining clusters for which the average similarity between members is the greatest. Each group is a vector comprised of 4 numbers (the percentages of amphibian, reptile, bird, and mammal families, or in the case of the Cenozoic for Madagascar, the percentages of clades belonging to each class known or inferred to have arrived independently).

Figure 4 shows a dendrogram of seven vertebrate assemblages (Madagascar during the Late Cretaceous, Paleogene, Neogene, and Quaternary, and the globe during the Late Cretaceous, Paleogene and Neogene). Each assemblage is represented by the percentages of tetrapod clades belonging to each of four classes: amphibians, reptiles, birds, and mammals. The dendrogram is specifically based on the Pearson correlations between these assemblages’ percent profiles. There are two main clusters – the Cretaceous and the Cenozoic assemblages. The first has only two members – the Cretaceous of Madagascar and the global Cretaceous. The second cluster comprises all of the Cenozoic assemblages including the Quaternary of Madagascar. Madagascar’s Quaternary tetrapod clades as well as its subsets of colonizers from the Paleogene and Neogene resemble (in percentages of amphibians, reptiles, birds, and mammals) corresponding global values, supporting the view that most of Madagascar’s Quaternary terrestrial vertebrates derive from transoceanic dispersal (and not vicariance) (Figure 4).

## Discussion and Conclusions

Our assessment has yielded insights into the factors that shaped the accumulation of Madagascar’s extant vertebrate fauna. Two events, (1) a mass extinction at the end of the Mesozoic and (2) a shift in ocean current direction in the mid-Miocene, have been particularly important. Whereas we tend to think of Madagascar as an isolated “sanctuary of nature”, it is perhaps better perceived as an experiment in differential extinction and filtration. As “filtration conditions” have changed over time (overlain on environmental change, shifts in tempo and mode of evolution, and the speciation and extinction of Malagasy and global vertebrate clades), the numbers and types of colonizers arriving from outside have correspondingly shifted.

Though there is little direct evidence, the K-T mass extinction likely affected Madagascar as it did the rest of the world. Very few vertebrate classes are represented in both the Mesozoic and the late Quaternary deposits of Madagascar. Only one low-level taxon represented in Mesozoic deposits (cf. *Erymnochelys*) is still represented in the extant vertebrate fauna of Madagascar. Of the vertebrate clades known (on the basis of fossil evidence) or inferred (on the basis of molecular evidence) to have arrived in the Mesozoic, only 37.1% are still extant, whereas over 90% of clades reconstructed as having arrived during the Cenozoic are still extant. Some of the Mesozoic clades may have survived the K-T event, but then succumbed to background clade extinction, but this is unlikely to account for the large difference in the taxonomic composition of the Mesozoic and Quaternary vertebrate faunas.

The striking difference between fishes and other vertebrates in percent survival across the K-T boundary requires explanation. We scored nine families of freshwater fish, three of which are known from Mesozoic deposits and are extinct, and six of which are inferred to have existed in the Mesozoic, but are still extant. This inferred clade survival rate (66.7%) is more than twice that of other vertebrate classes arriving in the Mesozoic. Two possible explanations should be considered. First, researchers studying responses of vertebrates to the asteroid impact and other insults (e.g., volcanic gasses, etc.) at the end of the Cretaceous have noted the unusual resilience of freshwater fishes (e.g., [146], [147]), suggesting that perhaps fishes were more protected from the insults that drove the extinction of more exposed, terrestrial vertebrates. Second, fish may have been less likely to have competitors arriving in the Cenozoic, unlike terrestrial and volant groups. Alternatively, it is possible that the extant freshwater fishes of Madagascar do not derive from Mesozoic ancestors in Madagascar but instead derive from colonizers in the early Cenozoic (see [51]); however this scenario would require crossing a large saltwater barrier. Even if only a few of the fish clades that we scored as descended from Mesozoic ancestors truly arrived, instead, during the early Cenozoic, this would greatly affect our inference regarding the vulnerability of fishes to extinction. We consider amphibians and freshwater fishes highly dispersal-disadvantaged, but this view may be wrong for fishes.

The shift in ocean current during the mid-Miocene affected the probability of successful colonization of different types of vertebrates. Early Cenozoic ocean conditions were conducive to rafting west-to-east from Africa to Madagascar by obligatorily terrestrial (dispersal-disadvantaged) forms, but as the Cenozoic progressed and both Madagascar and continental Africa drifted northward, dispersal-advantaged colonizers gained advantage over dispersal-disadvantaged colonizers. This bias became even more pronounced after the mid-Miocene change in prevailing ocean current direction from west-to-east to east-to-west [13].

What needs to be better understood is the dynamic of changes in Madagascar’s vertebrate biodiversity from the Mesozoic to the end of the Cenozoic (i.e., simultaneously examining the roles of colonization, and mass and background extinctions). The global diversity of tetrapod families is greater now than ever before, with shifts in the relative abundance of tetrapod classes over time [25], [26]. Madagascar had been isolated as an island for over 25 million years at the time of the K-T extinction. It was already distant from the nearest major landmasses with the Indian subcontinent far to the north. What is remarkable is that, despite Madagascar’s isolation since 25 million years prior to the K-T event, subsequent changes in the relative proportions of tetrapod classes on this island paralleled these changes elsewhere – amphibians dominate in the Paleozoic, reptiles in the Mesozoic, and birds and mammals in the Cenozoic, thus bearing testimony to a fundamental *lack* of isolation of Madagascar from the rest of the world. The relative proportions of Malagasy tetrapods belonging to each of these groups during the Mesozoic correlates strongly with global values, and the very different relative proportions of Malagasy Cenozoic colonizers correlate with the global values for relative abundance of tetrapod families during the Cenozoic.

Within volant groups, bats show a pattern strikingly different from that seen in birds. Malagasy bats arrive overwhelmingly from Africa (the closest source), while birds appear to have less constrained dispersal abilities, arriving from multiple geographic areas, some of which are quite distant. *Pteropus* is the one exception within Malagasy bats [148]; it is absent from the African mainland, and found on islands in the western Indian Ocean, including Madagascar, as well as Asia, the Indian subcontinent, and Australia [149]. Interestingly, *Pteropus* also inhabits the island of Pemba where it is separated from the African mainland by only 56 km [150].

While flight has clearly given bats a dispersal advantage compared to terrestrial animals, for most bat groups the power of flight does not appear to have conferred unconstrained dispersal abilities; distance apparently matters more to bats than it does to birds. Flight performance and aerodynamic flight efficiency appear to be limited by phylogenetic constraints; birds are acknowledged to fly longer distances, travel at faster speeds, and more often display migratory behavior than bats [151]. The perception that bats are able to disperse freely appears to have been based in part on the fact that many forms now recognized as distinct taxa [149] were historically grouped under one name, leading to artificial similarity of neighboring faunas. As stated by Andersen ([152], p. lxxvii), “the power of flight no doubt would enable a bat to spread over a much larger area than non-flying Mammalia, but, as a matter of fact, only in a very few cases is there any reason to believe that it has caused it to do so.” Further studies examining the different constraints on bat vs. bird dispersal, as well as the role of storms and cyclonic winds into these events, will yield important insights into this discussion.

In summary, we have shown that the biodiversity of tetrapod colonizers on Madagascar reflects that of the major vertebrate groups on other landmasses, shifting from reptiles to birds and mammals during the transition from the Mesozoic to the Cenozoic. The shift in class composition of arriving clade ancestors after the K-T boundary underscores the importance of overwater dispersal in shaping Madagascar’s extant biodiversity. Madagascar is not simply an isolated island harboring a relict, reptile-rich fauna, but rather it reflects a shift in relative vertebrate taxonomic diversity in temporal synchrony with the rest of the globe. Whereas extant Malagasy fishes appear to derive from Mesozoic ancestors, all extant mammals and almost 95% of extant bird clades derive from Cenozoic colonizers. Two-thirds to four-fifths of extant amphibian and reptile clades are Cenozoic in origin. Madagascar’s vertebrate fauna has been shaped by a combination of vicariance and dispersal, but dispersal explains the presence of the majority of the island’s extant vertebrate fauna.

## Supporting Information

Table S1 **Database summarizing biogeographic scenarios of Madagascar’s vertebrate fauna.** Class was scored as 1 = Osteichthyes, 2 = Amphibia, 3 = Reptilia, 4 = Aves, 5 = Mammalia. Animals were scored as 1 = extinct or 2 = extant. Time was scored as 1 = Pre K-T, 2 = Post K-T to mid-Miocene, and 3 = mid Miocene to present. Source was scored as 1 = Gondwana, 2 = Indo-Madagascar, 3 = Africa, 4 = India or SE Asia. Malagasy fossil record was scored as 1 = none, 2 = Mesozoic, 3 = Quaternary. Type of ancestor was scored as 1 = obligate freshwater, 2 = terrestrial, 3 = facultative swimmer, 4 = volant. Ocean current direction at time of arrival was coded as 1 = favoring dispersal from Africa, 2 = favoring dispersal from Asia. Dispersal ability was coded as 1 = dispersal-disadvantaged (obligate freshwater or terrestrial), 2 = dispersal-advantaged (facultative swimmer or volant). Exceptions to these categorizations of dispersal-advantaged and disadvantaged taxa are provided in footnotes.
(DOCX)
