# Supplementary material for: Imperfect Isolation: Factors and Filters Shaping Madagascar’s Extant Vertebrate Fauna
Source: PLoS One. 2013 Apr 23;8(4):e62086. doi: 10.1371/journal.pone.0062086 (PMC3633922; doi:10.1371/journal.pone.0062086)
Supplement: Table S1 — Database summarizing biogeographic scenarios of Madagascar’s vertebrate fauna. Class was scored as 1 = Osteichthyes, 2 = Amphibia, 3 = Reptilia, 4 = Aves, 5 = Mammalia. Animals were scored as 1 = extinct or 2 = extant. Time was scored as 1 = Pre K-T, 2 = Post K-T to mid-Miocene, and 3 = mid Miocene to present. Source was scored as 1 = Gondwana, 2 = Indo-Madagascar, 3 = Africa, 4 = India or SE Asia. Malagasy fossil record was scored as 1 = none, 2 = Mesozoic, 3 = Quaternary. Type of ancestor was scored as 1 = obligate freshwater, 2 = terrestrial, 3 = facultative swimmer, 4 = volant. Ocean current direction at time of arrival was coded as 1 = favoring dispersal from Africa, 2 = favoring dispersal from Asia. Dispersal ability was coded as 1 = dispersal-disadvantaged (obligate freshwater or terrestrial), 2 = dispersal-advantaged (facultative swimmer or volant). Exceptions to these categorizations of dispersal-advantaged and disadvantaged taxa are provided in footnotes. (DOCX) [file pone.0062086.s001.docx]

**Table S1.**

| **Taxon**  **(Ancestor of)** | **Malagasy Genera** | **Class** | **Extinct / Extant** | **Time** | **Source** | **Malagasy fossil record** | **Type of ancestor** | **Current**  **Direction** | **Dispersal Ability** | **References** |
| --- | --- | --- | --- | --- | --- | --- | --- | --- | --- | --- |
| Aplocheilidae | *Pachypanchax* | 1 | 2 | 1 | 2 | 1 | 1 | . | 1 | [1] |
| Bedotiidae | *Bedotia*, *Rheocles* | 1 | 2 | 1 | 1 | 1 | 1 | . | 1 | [2] |
| Cichlidae | *Oxylapia, Paratilapia, Paretroplus, Ptychochromis, Ptychochromoides* | 1 | 2 | 1 | 1 | 1 | 1 | . | 1 | [3-9] |
| Clupeidae | *Sauvagella, Spratellomorpha* | 1 | 2 | 1 | 1 | 1 | 1 | . | 1 | [4,10] |
| Milyeringidae | *Typhleotris* | 1 | 2 | 1 | 1 | 1 | 1 | . | 1 | [11] |
| Lepisosteidae | *Lepisosteus* | 1 | 1 | 1 | 1 | 2 | 1 | . | 1 | [12] |
| Mugilidae | *Agonostomus, Liza, Mugil* | 1 | 2 | 1 | 1 | 1 | 1 | . | 1 | [4] |
| Phyllodontidae | cf. *Egertonia* sp. | 1 | 1 | 1 | 1 | 2 | 1 | . | 1 | [12] |
| Pycnodontidae | cf. *Coelodus* sp. | 1 | 1 | 1 | 1 | 2 | 1 | . | 1 | [13] |
| Ceratophryinae | *Beelzebufo* | 2 | 1 | 1 | 1 | 2 | 2 | . | 1 | [14] |
| Hyperoliidae | *Heterixalus* | 2 | 2 | 2 | 3 | 1 | 2 | 1 | 1 | [15] |
| Microhylidae | Cophylinae + Scaphiophryninae | 2 | 2 | 1 | - | 1 | 2 | . | 1 | [16] |
| Microhylidae | Dyscophinae | 2 | 2 | 2 | 4 | 1 | 2 | 1 | 1 | [16] |
| Mantellidae | *Aglyptodactylus*, *Blommersia*, *Boehmantis*, *Boophis*, *Gephyromantis*, *Guibemantis*, *Laliostoma*, *Mantella*, *Mantidactylus*, *Spinomantis*, *Tsingymantis*, *Wakea* | 2 | 2 | 2 | 4 | 1 | 2 | 1 | 1 | [16-22] |
| Ptychadenidae | *Ptychadena* | 2 | 2 | 3 | 3 | 1 | 2 | 2 | 2 | [23,24] |
| Bothremydidae | Kinkonychelys | 3 | 1 | 1 | 1 | 2 | 3 | . | 2 | [25] |
| Podocnemidae | Erymnochelys | 3 | 2 | 1 | 1 | 2 | 3 | . | 2 | [26,27] |
| Testudinae | *Astrochelys, Aldabrachelys* | 3 | 2 | 3 | 3 | 3 | 3 | 2 | 2 | [28] |
| Crocodylidae | *Crocodylus* | 3 | 2 | 3 | 3 | 3 | 3 | 2 | 2 | [29] |
| Crocodylidae | *Voay* | 3 | 1 | 3 | 3 | 3 | 3 | 2 | 2 | [29,30] |
| Mahajangasuchidae | *Mahajangasuchus* | 3 | 1 | 1 | 1 | 2 | 3 | . | 2 | [31,32] |
| Notosuchia | *Araripesuchus* | 3 | 1 | 1 | 1 | 2 | 3 | . | 2 | [33] |
| Notosuchia | *Simosuchus* | 3 | 1 | 1 | 1 | 2 | 3 | . | 2 | [34,35] |
| Trematochampsidae | *Miadanasuchus* | 3 | 1 | 1 | 1 | 2 | 3 | . | 2 | [36] |
| Chamaeleonidae | *Brookesia*, *Calumma*, *Furcifer* | 3 | 2 | 2 | 2 | 1 | 2 | 1 | 1 | [37] |
| ?Cordylidae | *Konkasaurus* | 3 | 1 | 1 | 1 | 2 | 2 | . | 1 | [38] |
| Dromaeosauridae | *Rahonavis* | 3 | 1 | 1 | 1 | 2 | 4 | . | 2 | [39,40] |
| Gerrhosauridae | *Zonosaurus, Tracheloptychus* | 3 | 2 | 1 | 3 | 1 | 2 | . | 1 | [41] |
| Opluridae | *Chalarodon, Oplurus* | 3 | 2 | 1 | 1 | 1 | 2 | . | 1 | [27] |
| Gekkonidae^^[[1]](#footnote-1)^^ | *Blaesodactylus* | 3 | 2 | 2 | 3 | 1 | 2 | 1 | 2 | [42] |
| Gekkonidae^1^ | *Hemidactylus* mercatorius | 3 | 2 | 3 | 3 | 1 | 2 | 2 | 2 | [42] |
| Gekkonidae^1^ | *Hemidactylus* playcephalus | 3 | 2 | 3 | 3 | 1 | 2 | 2 | 2 | [42] |
| Scincidae | *Trachylepis* | 3 | 2 | 2 | 3 | 1 | 2 | 1 | 1 | [43] |
| Scincidae^^[[2]](#footnote-2)^^ | Cryptoblepharus | 3 | 2 | 2 | 4 | 1 | 2 | 1 | 2 | [44] |
| Scincidae | Amphiglossus, Androngo, Madascincus, Paracontias, Pseudoacontias, Pygomeles, Sirenoscincus, Voeltzkowia | 3 | 2 | 2 | 3 | 1 | 2 | 1 | 1 | [45,46] |
| Boidae | *Acrantophis*, *Sanzinia*, *Calabaria* | 3 | 2 | 1 | 1 | 1 | 2 | . | 1 | [27] |
| Lamprophiidae | *Alluaudina*,  *Compsophis*,  *Dromicodryas*,  *Geodipsas*,  *Heteroliodon*,  *Ithycyphus*, *Langaha*,  *Leioheterodon*,  *Liophidium*,  *Liopholidophis*,  *Madagascarophis*,  *Micropisthodon*,  *Pseudoxyrhopus*,  *Stenophis* | 3 | 2 | 2 | 3 | 1 | 2 | 1 | 1 | [47] |
| Lamprophiidae | *Mimophis* | 3 | 2 | 3 | 3 | 1 | 2 | 2 | 1 | [47] |
| Madtsoiidae | *Madtsoia* | 3 | 1 | 1 | 1 | 2 | 2 | . | 1 | [48] |
| Madtsoiidae | *Menarana* | 3 | 1 | 1 | 1 | 2 | 2 | . | 1 | [49] |
| Nigerophiidae | *Kelyophis* | 3 | 1 | 1 | 1 | 2 | 2 | . | 1 | [49] |
| Typhlopidae | *Typhlops* | 3 | 2 | 2 | 1 | 1 | 2 | 1 | 1 | [50] |
| Xenotyphlopidae | *Xenotyphlops* | 3 | 2 | 1 | 1 | 1 | 2 | . | 1 | [50] |
| Abelisauridae | *Majungasaurus* | 3 | 1 | 1 | 1 | 2 | 2 | . | 1 | [51] |
| Noasauridae | *Masiakasaurus* | 3 | 1 | 1 | 1 | 2 | 2 | . | 1 | [52] |
| Nemegtosauridae | *Rapetosaurus* | 3 | 1 | 1 | 1 | 2 | 2 | . | 1 | [53] |
| Aepyornithidae | *Aepyornis, Mullerornis* | 4 | 1 | 1 | 1 | 3 | 2 | . | 1 | [54-56] |
| Apodidae | *Aerodramus* | 4 | 2 | 3 | 4 | 1 | 4 | 2 | 2 | [57] |
| Bernieridae | *Thamnornis, Xanthomixis* | 4 | 2 | 2 | 3 | 1 | 4 | 1 | 2 | [58] |
| Campephagidae | *Coracina* | 4 | 2 | 2 | 4 | 1 | 4 | 1 | 2 | [59,60] |
| Columbidae | *Alectroeanas* | 4 | 2 | 2 | 4 | 1 | 4 | 1 | 2 | [61] |
| Dicruridae | *Dicrurus* | 4 | 2 | 3 | 3 | 1 | 4 | 2 | 2 | [62] |
| Eurylaimidae | *Neodrepanis, Philepitta* | 4 | 2 | 2 | 3 | 1 | 4 | 1 | 2 | [58] |
| Mesitornithidae | *Mesitornis, Monias* | 4 | 2 | 1 | 1 | 3 | 2 | . | 1 | [54,63] |
| Motacillidae | *Motacilla* | 4 | 2 | 3 | 3 | 1 | 4 | 2 | 2 | [64] |
| Nectarinidae | *Nectarinia* *souimanga* clade | 4 | 2 | 3 | 3 | 1 | 4 | 2 | 2 | [65] |
| Nectarinidae | *Nectarinia* *notata* clade | 4 | 2 | 3 | 3 | 1 | 4 | 2 | 2 | [65] |
| Ornithurae | *Vorona* | 4 | 1 | 1 | 1 | 2 | 4 | . | 2 | [66] |
| Psittacidae | *Agapornis* | 4 | 2 | 2 | 4 | 3 | 4 | 1 | 2 | [54,67] |
| Psittacidae | *Coracopsis* | 4 | 2 | 2 | 4 | 3 | 4 | 1 | 2 | [54,67,68] |
| Pycnonotidae | *Hypsipetes* | 4 | 2 | 3 | 4 | 3 | 4 | 2 | 2 | [54,69] |
| Strigidae | *Otus* | 4 | 2 | 3 | 4 | 3 | 4 | 2 | 2 | [54,60] |
| Sturnidae | *Hartlaubius* | 4 | 2 | 3 | 4 | 1 | 4 | 2 | 2 | [70] |
| Vangidae | *Artamella*, *Calicalicus*, *Cyanolanius*, *Euryceros*, *Falculea*, *Hypositta*, *Leptopterus*, *Mystacornis*, *Newtonia*, *Oriolia*, *Pseudobias*, *Schetba*, *Tylas*, *Xenopirostris*, *Vanga* | 4 | 2 | 2 | 3 | 1 | 4 | 1 | 2 | [71] |
| Zosteropidae | *Zosterops* *borbonicus* lineage | 4 | 2 | 3 | 4 | 1 | 4 | 2 | 2 | [72] |
| Zosteropidae | *Zosterops* *maderaspatanus* lineage | 4 | 2 | 3 | 4 | 1 | 4 | 2 | 2 | [72] |
| Bibymalagasia | *Plesiorycteropus* | 5 | 1 | 2 | 3 | 3 | 2 | 1 | 1 | [73-75] |
| Emballonuridae | *Paremballonura atrata, P. tiavato* | 5 | 2 | 2 | 3 | 1 | 4 | 1 | 2 | [76,77] |
| Emballonuridae | *Coleura kibomalandy* | 5 | 2 | 2 | 3 | 1 | 4 | 1 | 2 | [76-78] |
| Emballonuridae | *Taphozous mauritianus* | 5 | 2 | 2 | 3 | 1 | 4 | 1 | 2 | [76,78] |
| Eupleridae | *Cryptoprocta, Eupleres, Fossa, Galidia, Galidictis* | 5 | 2 | 2 | 3 | 3 | 2 | 1 | 1 | [79,80] |
| Hippopotamidae | *Hippopotamus lemerlei, H. laloumena* | 5 | 1 | 3 | 3 | 3 | 3 | 2 | 2 | [81,82] |
| Hippopotamidae | *Hexaprotodon guldbergi* | 5 | 1 | 3 | 3 | 3 | 3 | 2 | 2 | [81-83] |
| Hipposideridae | *Hipposideros* | 5 | 2 | 3 | 3 | 3 | 4 | 2 | 2 | [76,84] |
| Hipposideridae | *Triaenops* (*T. auritus* + *T. furculus + T. goodmani*) | 5 | 2 | 3 | 3 | 3 | 4 | 2 | 2 | [84,85] |
| Hipposideridae | *Triaenops* (*T. menamena)* | 5 | 2 | 3 | 3 | 1 | 4 | 2 | 2 | [85] |
| Lemuroidea | *Cheirogaleus, Daubentonia, Eulemur, Hapalemur, Lemur, Lepilemur, Microcebus, Mirza, Propithecus, Varecia* | 5 | 2 | 2 | 3 | 3 | 2 | 1 | 1 | [86,87] |
| Marsupialia | Genus indet. | 5 | 1 | 1 | 1 | 2 | 2 | . | 1 | [88] |
| Molossidae | *Chaerephon leucogaster* | 5 | 2 | 3 | 3 | 1 | 4 | 2 | 2 | [78,89,90] |
| Molossidae | *Chaerephon atsinanana* | 5 | 2 | 3 | 3 | 1 | 4 | 2 | 2 | [78,90] |
| Molossidae | *Chaerephon jobimena* | 5 | 2 | 3 | 3 | 1 | 4 | 2 | 2 | [78,90] |
| Molossidae | *Mops leucostigma* | 5 | 2 | 3 | 3 | 1 | 4 | 2 | 2 | [78,90] |
| Molossidae | *Mops midas* | 5 | 2 | 3 | 3 | 1 | 4 | 2 | 2 | [78,90] |
| Molossidae | *Mormopterus jugularis* | 5 | 2 | - | - | 3 | 4 | . | 2 | [78,80,90] |
| Molossidae | *Otomops madagascariensis* | 5 | 2 | - | - | 3 | 4 | . | 2 | [78,80,90] |
| Molossidae | *Tadarida fulminans* | 5 | 2 | 3 | 3 | 1 | 4 | 2 | 2 | [78] |
| Multituberculata | Genus indet. | 5 | 1 | 1 | 1 | 2 | 2 | . | 1 | [91] |
| Myzopodidae | *Myzopoda* | 5 | 2 | 2 | - | 1 | 4 | 1 | 2 | [76] |
| Nesomyinae | *Brachytarsomys, Eliurus, Hypogeomys, Macrotarsomys* | 5 | 2 | 2 | 3 | 3 | 2 | 1 | 1 | [79,92] |
| Nycteridae | *Nycteris* | 5 | 2 | 2 | 3 | 1 | 4 | 1 | 2 | [76] |
| Pteropodidae | *Pteropus rufus* | 5 | 2 | 3 | 4 | 3 | 4 | 2 | 2 | [84,93] |
| Pteropodidae | *Eidolon dupreanum* | 5 | 2 | - | 3 | 3 | 4 | . | 2 | [76,84] |
| Pteropodidae | *Rousettus madagascariensis* | 5 | 2 | 3 | - | 3 | 4 | 2 | 2 | [84,94] |
| Sudamericidae | *Lavanify* | 5 | 1 | 1 | 1 | 2 | 2 | . | 1 | [95] |
| Tenrecidae | *Limnogale, Microgale, Oryzorictes, Echinops, Setifer, Hemicentetes, Tenrec* | 5 | 2 | 2 | 3 | 3 | 2 | 1 | 1 | [80,96] |
| Vespertilionidae | *Myotis* | 5 | 2 | 3 | 3 | 3 | 4 | 2 | 2 | [76,84,97] |
| Vespertilionidae | *Scotophilus marovaza* | 5 | 2 | 3 | 3 | 1 | 4 | 2 | 2 | [98] |
| Vespertilionidae | *Scotophilus robustus* | 5 | 2 | 3 | 3 | 1 | 4 | 2 | 2 | [98] |
| Vespertilionidae | *Neoromica matroka* | 5 | 2 | 3 | 3 | 1 | 4 | 2 | 2 | [78] |

**REFERENCES S1**

1. Murphy WJ, Collier GE (1997) A molecular phylogeny for aplocheiloid fishes (Atherinomorpha, Cyprinodontiformes): The role of vicariance and the origins of annualism. Molecular Biology and Evolution 14: 790-799.

2. Sparks JS, Smith WL (2004) Phylogeny and biogeography of the Malagasy and Australian rainbowfishes (Teleostei: Melanotaenioidei): Gondwanan vicariance and evolution in freshwater. Molecular Phylogenetics and Evolution 33: 719-734.

3. Stiassny MLJ (1992) Phylogenetic analysis and the role of systematics in the biodiversity crisis. In: Eldredge N, editor. Systematics, Ecology and the Biodiversity Crisis. New York: Columbia University Press. pp. 109-120.

4. Benstead JP, De Rham PH, Gattolliat JL, Gibon FM, Loiselle PV, et al. (2003) Conserving Madagascar's freshwater biodiversity. Bioscience 53: 1101-1111.

5. Briggs JC (2003) Fishes and birds: Gondwana life rafts reconsidered. Systematic Biology 52: 548-553.

6. Chakrabarty P (2004) Cichlid biogeography: comment and review. Fish and Fisheries 5: 97-119.

7. Sparks JS (2004) Molecular phylogeny and biogeography of the Malagasy and South Asian cichlids (Teleostei : Perciformes : Cichlidae). Molecular Phylogenetics and Evolution 30: 599-614.

8. Sparks JS, Smith WL (2004) Phylogeny and biogeography of cichlid fishes (Teleostei: Perciformes: Cichlidae). Cladistics 20: 501-517.

9. Sparks JS, Smith WL (2005) Freshwater fishes, dispersal ability, and nonevidence: "Gondwana Life Rafts" to the rescue. Systematic Biology 54: 158-165.

10. Stiassny MLJ (2002) Revision of *Sauvagella* Bertin (Clupeidae; Pellonulinae; Ehiravini) with a description of a new species from the freshwaters of Madagascar and diagnosis of the Ehiravini. Copeia: 67-76.

11. Sparks JS, Chakrabarty P (2012) Revision of the endemic Malagasy cavefish genus *Typhleotris* (Teleostei: Gobiiformes: Milyeringidae), with discussion of its phylogenetic placement and description of a new species. American Museum Novitates 3764: 1-28.

12. Gottfried M, Krause D (1998) First record of gars (Lepisosteidae, Actinopterygii) on Madagascar: Late Cretaceous remains from the Mahajanga Basin. Journal of Vertebrate Paleontology 18: 275-279.

13. Rogers RR, Hartman JH, Krause DW (2000) Stratigraphic analysis of Upper Cretaceous rocks in the Mahajanga Basin, northwestern Madagascar: Implications for ancient and modern faunas. The Journal of Geology 108: 275-301.

14. Evans SE, Jones MEH, Krause DW (2008) A giant frog with South American affinities from the Late Cretaceous of Madagascar. Proceedings of the National Academy of Sciences, USA 105: 2951-2956.

15. Vences M, Kosuch J, Glaw F, Bohme W, Veith M (2003) Molecular phylogeny of hyperoliid treefrogs: biogeographic origin of Malagasy and Seychellean taxa and re-analysis of familial paraphyly. Journal of Zoological Systematics and Evolutionary Research 41: 205-215.

16. van der Meijden A, Vences M, Hoegg S, Boistel R, Channing A, et al. (2007) Nuclear gene phylogeny of narrow-mouthed toads (Family: Microhylidae) and a discussion of competing hypotheses concerning their biogeographical origins. Molecular Phylogenetics and Evolution 44: 1017-1030.

17. Bossuyt F, Milinkovitch MC (2001) Amphibians as indicators of early tertiary "out-of-India" dispersal of vertebrates. Science 292: 93-95.

18. Vences MJ, Glaw F (2001) When molecules claim for taxonomic changes: new proposals on the classification of Old World treefrogs. Spixiana 24: 85-92.

19. Vences M, Vieites DR, Glaw F, Brinkmann H, Kosuch J, et al. (2003) Multiple overseas dispersal in amphibians. Proceedings of the Royal Society of London Series B-Biological Sciences 270: 2435-2442.

20. Bossuyt F, Brown RM, Hillis DM, Cannatella DC, Milinkovitch MC (2006) Phylogeny and biogeography of a cosmopolitan frog radiation: Late Cretaceous diversification resulted in continent-scale endemism in the family ranidae. Systematic Biology 55: 579-594.

21. Van Bocxlaer I, Roelants K, Biju SD, Nagaraju J, Bossuyt F (2006) Late Cretaceous vicariance in Gondwanan amphibians. PLoS ONE 1: 6.

22. Kurabayashi A, Sumida M, Yonekawa H, Glaw F, Vences M, et al. (2008) Phylogeny, recombination, and mechanisms of stepwise mitochondrial genome reorganization in mantellid frogs from Madagascar. Molecular Biology and Evolution 25: 874-891.

23. Vences M, Kosuch J, Rodel MO, Lotters S, Channing A, et al. (2004) Phylogeography of *Ptychadena mascareniensis* suggests transoceanic dispersal in a widespread African-Malagasy frog lineage. Journal of Biogeography 31: 593-601.

24. Measey GJ, Vences M, Drewes RC, Chiari Y, Melo M, et al. (2007) Freshwater paths across the ocean: molecular phylogeny of the frog *Ptychadena newtoni* gives insights into amphibian colonization of oceanic islands. Journal of Biogeography 34: 7-20.

25. Gaffney ES, Krause DW, Zalmout IS (2009) *Kinkonychelys*, a new side-necked turtle (Pelomedusoides: Bothremydidae) from the Late Cretaceous of Madagascar. American Museum Novitates 3662: 1-25.

26. Gaffney E, Forster C (2003) Side-necked turtle lower jaws (Podocnemididae, Bothremydidae) from the Late Cretaceous Maevarano Formation of Madagascar. American Museum Novitates: 3397: 1-13.

27. Noonan BP, Chippendale PT (2006) Vicariant origin of Malagasy reptiles supports Late Cretaceous Antarctic landbridge. American Naturalist 168: 730-741.

28. Palkovacs EP, Gerlach J, Caccone A (2002) The evolutionary origin of Indian Ocean tortoises (*Dipsochelys*). Molecular Phylogenetics and Evolution 24: 216-227.

29. Brochu CA (2007) Morphology, relationships, and biogeographical significance of an extinct horned crocodile (Crocodylia, Crocodylidae) from the Quaternary of Madagascar. Zoological Journal of the Linnean Society 150: 835-863.

30. Bickelmann C, Klein N (2009) The late Pleistocene horned crocodile *Voay robustus* (Grandidier & Vaillant, 1872) from Madagascar in the Museum fur Naturkunde Berlin. Fossil Record 12: 13-21.

31. Buckley G, Brochu C (1999) An enigmatic new crocodile from the Upper Cretaceous of Madagascar. Special Papers in Paleontology 60: 149-175.

32. Turner AH, Buckley GA (2008) *Mahajangasuchus insignis* (Crocodyliformes: Mesoeucrocodylia) cranial anatomy and new data on the origin of the eusuchian-style palate. Journal of Vertebrate Paleontology 28: 382-408.

33. Turner AH (2006) Osteology and phylogeny of a new species of Araripesuchus (Crocodyliformes: Mesoeucrocodylia) from the Late Cretaceous of Madagascar. Historical Biology 18: 255-369.

34. Buckley G, Brochu C, Krause D, Pol D (2000) A pug-nosed crocodyliform from the Late Cretaceous of Madagascar. Nature 405: 941-944.

35. Turner AH, Sertich JJW (2010) Phylogenetic history of *Simosuchus clarki* (Crocodyliformes: Notosuchia) from the Late Cretaceous of Madagascar. Journal of Vertebrate Paleontology 30: 177-236.

36. Rasmusson Simons EL, Buckley GA (2009) New material of "*Trematochampsa*" *oblita* (Crocodyliformes,Trematochampsidae) from the Late Cretaceous of Madagascar. Journal of Vertebrate Paleontology 29: 599-604.

37. Raxworthy C, Forstner M, Nussbaum R (2002) Chameleon radiation by oceanic dispersal. Nature 415: 784-787.

38. Krause DW, Evans SE, Gao K-Q (2003) First definitive record of Mesozoic lizards from Madagascar. Journal of Vertebrate Paleontology 23: 842-856.

39. Forster C (1999) Gondwanan dinosaur evolution and biogeographic analysis. Journal of African Earth Sciences 28: 169-185.

40. Makovicky PJ, Apestegula S, Agnolín, FL (2005) The earliest droeaeosaurid theropod from South America. Nature 437: 1007-1011.

41. Raselimanana AP, Noonan B, Karanth KP, Gauthier J, Yoder AD (2009) Phylogeny and evolution of Malagasy plated lizards. Molecular Phylogenetics and Evolution 50: 336-344.

42. Vences M, Wanke S, Vieites DR, Branch WR, Glaw F, et al. (2004) Natural colonization or introduction? Phylogeographical relationships and morphological differentiation of house geckos (*Hemidactylus*) from Madagascar. Biological Journal of the Linnean Society 83: 115-130.

43. Mausfeld P, Vences M, Schmitz A, Veith M (2000) First data on the molecular phylogeography of scincid lizards of the genus *Mabuya*. Molecular Phylogenetics and Evolution 17: 11-14.

44. Rocha S, Carretero MA, Vences M, Glaw F, Harris DJ (2006) Deciphering patterns of transoceanic dispersal: the evolutionary origin and biogeography of coastal lizards (*Cryptoblepharus*) in the Western Indian Ocean region. Journal of Biogeography 33: 13-22.

45. Brandley M, Huelsenbeck J, Wiens J (2008) Rates and patterns in the evolution of snake-like body form in squamate reptiles: Evidence for repeated re-evolution of lost digits and long-term persistence of intermediate body forms. Evolution 62: 2042-2064.

46. Crottini A, Dordel J, Köhler J, Glaw F, Schmitz A, et al. (2009) A multilocus phylogeny of Malagasy scincid lizards elucidates the relationships of the fossorial genera *Androngo* and *Cryptoscincus*. Molecular Phylogenetics and Evolution 53: 345-350.

47. Nagy Z, Joger U, Wink M, Glaw F, Vences M (2003) Multiple colonization of Madagascar and Socotra by colubrid snakes: evidence from nuclear and mitochondrial gene phylogenies. Proceedings of the Royal Society of London 270: 2613-2621.

48. Hoffstetter R (1961) Nouveaux restes d'un serpent boïdé (Madtsoia madagascariensis nov. sp.) dans le Crétacé supérieur de Madagascar. Bulletin du Muséum national d'Histoire naturelle, Paris 33: 152-160.

49. LaDuke TC, Krause DW, Scanlon JD, Kley NJ (2010) A Late Cretaceous (Maastrichtian) snake assemblage from the Maevarano Formation, Mahajanga Basin, Madagascar. Journal of Vertebrate Paleontology 30: 109-138.

50. Vidal N, Marin J, Morini M, Donnellan S, Branch W, et al. (2010) Blindsnake evolutionary tree reveals long history on Gondwana. Biology Letters doi: 10.1098/rsbl.2010.0220.

51. Krause DW, Sampson SD, Carrano MT, O'Connor PM (2007) Overview of the history of discovery, taxonomy, phylogeny, and biogeography of *Majungasaurus crenatissimus* (Theropoda: Abelisauridae) from the Late Cretaceous of Madagascar. In: Sampson SD, Krause DW, editors. *Majungasaurus crenatissimus* (Theropoda: Abelisauridae) from the Late Cretaceous of Madagascar: Society of Vertebrate Paleontology Memoir. pp. 1-20.

52. Sampson S, Carrano M, Forster C (2001) A bizarre predatory dinosaur from the Late Cretaceous of Madagascar. Nature 409: 504-506.

53. Curry-Rogers K, Forster C (2001) The last of the dinosaur titans: a new sauropod from Madagascar. Nature 412: 530-534.

54. Burney DA, James HF, Grady FV, Jean-Gervais Rafamantanantsoa R, Wright HT, et al. (1997) Environmental change, extinction and human activity: evidence from caves in NW Madagascar. Journal of Biogeography 24: 755-767.

55. Cooper A, Lalueza-Fox C, Anderson S, Rambaut A, Austin J, et al. (2001) Complete mitochondrial genome sequences of two extinct moas clarify ratite evolution. Nature 409: 704-707.

56. Bourdon E, de Ricqles A, Cubo J (2009) A new Transantarctic relationship: morphological evidence for a Rheidae–Dromaiidae–Casuariidae clade (Aves, Palaeognathae, Ratitae). Zoological Journal of the Linnean Society 156: 641-663.

57. Johnson K, Clayton D (1999) Swiftlets on islands: genetics and phylogeny of the Seychelles and Mascarene swiftlets. Phelsuma 7: 9-13.

58. Beresford P, Barker FK, Ryan PG, Crowe TM (2005) African endemics span the tree of songbirds (Passeri): molecular systematics of several evolutionary 'enigmas'. Proceedings of the Royal Society B-Biological Sciences 272: 849-858.

59. Fuchs J, Cruaud C, Couloux A, Pasquet E (2007) Complex biogeographic history of the cuckoo-shrikes and allies (Passeriformes : Campephagidae) revealed by mitochondrial and nuclear sequence data. Molecular Phylogenetics and Evolution 44: 138-153.

60. Fuchs J, Pons JM, Goodman SM, Bretagnolle V, Melo M, et al. (2008) Tracing the colonization history of the Indian Ocean scops-owls (Strigiformes: *Otus*) with further insight into the spatio-temporal origin of the Malagasy avifauna. Bmc Evolutionary Biology 8: 197.

61. Shapiro B, Sibthorpe D, Rambaut A, Austin J, Wragg GM, et al. (2002) Flight of the dodo. Science 295: 1683-1683.

62. Pasquet E, Pons JM, Fuchs J, Cruaud C, Bretagnolle V (2007) Evolutionary history and biogeography of the drongos (Dicruridae), a tropical Old World clade of corvoid passerines. Molecular Phylogenetics and Evolution 45: 158-167.

63. Fain MG, Houde P (2004) Parallel radiations in the primary clades of birds. Evolution 58: 2558-2573.

64. Voelker G (2002) Systematics and historical biogeography of wagtails: dispersal versus vicariance revisited. Condor 104: 725-739.

65. Warren BH, Bermingham E, Bowie RCK, Prys-Jones RP, Thebaud C (2003) Molecular phylogeography reveals island colonization history and diversification of western Indian Ocean sunbirds (*Nectarinia*: Nectariniidae). Molecular Phylogenetics and Evolution 29: 67-85.

66. Forster CA, Chiappe LM, Krause DW, Sampson SD (1996) The first Cretaceous bird from Madagascar. Nature 382: 532-534.

67. Schweizer M, Seehausen O, Guntert M, Hertwig ST (2010) The evolutionary diversification of parrots supports a taxon pulse model with multiple trans-oceanic dispersal events and local radiations. Molecular Phylogenetics and Evolution 54: 984-994.

68. Wright TF, Schirtzinger EE, Matsumoto T, Eberhard JR, Graves GR, et al. (2008) A multilocus molecular phylogeny of the parrots (Psittaciformes): support for a Gondwanan origin during the Cretaceous. Molecular Biology and Evolution 25: 2141-2156.

69. Warren BH, Bermingham E, Prys-Jones RP, Thebaud C (2005) Tracking island colonization history and phenotypic shifts in Indian Ocean bulbuls (*Hypsipetes*: Pycnonotidae). Biological Journal of the Linnean Society 85: 271-287.

70. Zuccon D, Cibois A, Pasquet E, Ericson PGP (2006) Nuclear and mitochondrial sequence data reveal the major lineages of starlings, mynas and related taxa. Molecular Phylogenetics and Evolution 41: 333-344.

71. Reddy S, Driskell A, Rabosky DL, Hackett SJ, Schulenberg TS (2012) Diversification and the adaptive radiation of the vangas of Madagascar. Proceedings of the Royal Society B.

72. Warren BH, Bermingham E, Prys-Jones RP, Thebaud C (2006) Immigration, species radiation and extinction in a highly diverse songbird lineage: white-eyes on Indian Ocean islands. Molecular Ecology 15: 3769-3786.

73. MacPhee RDE (1994) Morphology, adaptations, and relationships of *Plesiorycteropus*, and a diagnosis of a new order of eutherian mammals. Bulletin of the American Museum of Natural History 220: 1-214.

74. Asher R, Novacek M, Geisler J (2003) Relationships of endemic African mammals and their fossil relatives based on morphological and molecular evidence. Journal of Mammalian Evolution 10: 131-194.

75. Horovitz I (2004) Eutherian mammal systematics and the origins of South American ungulates as based on postcranial osteology. In: Dawson MR, Lillegraven JA, editors. Fanfare for an Uncommon Paleontologist: Papers in Honor of Malcolm C McKenna: Bulletin of the Carnegie Museum of Natural History. pp. 63-79.

76. Teeling EC, Springer MS, Madsen O, Bates P, O'Brien SJ, et al. (2005) A molecular phylogeny for bats illuminates biogeography and the fossil record. Science 307: 580-584.

77. Goodman SM, Puechmaille SJ, Friedli-Weyeneth N, Gerlach J, Ruedi M, et al. Phylogeny of the tride Emballonurini (Family Emballonuridae) with descriptions of a new genus for Malagasy *Emballonura* and a new species of *Coleura*.

78. Goodman SM (2011) Les chauves-souris de Madagascar. Antananarivo: Association Vahatra. 129 p.

79. Poux C, Madsen O, Marquard E, Vieites DR, de Jong WW, et al. (2005) Asynchronous colonization of Madagascar by the four endemic clades of primates, tenrecs, carnivores, and rodents as inferred from nuclear genes. Systematic Biology 54: 719-730.

80. Muldoon KM, DeBlieux DD, Simons EL, Chatrath PS (2009) The subfossil occurrence and paleoecological significance of small mammals at Ankilitelo Cave, southwestern Madagascar. Journal of Mammalogy 90: 26-55.

81. Stuenes S (1989) Taxonomy, habits and relationships of the sub-fossil Madagascan hippopotamuses, *Hippopotamus lemerlei* and *H. madagascariensis*. Journal of Vertebrate Paleontology 9: 241-268.

82. Fovet W, Faure M, Guérin, C (2011) *Hippopotamus guldbergi* n. sp.: révision du statut d'*Hippopotamus madagascariensis* Guldberg, 1883, après plus d'un siècle de malentendus et de confusions taxonomiques. Zoosystema 33: 61-82.

83. Boisserie J-R (2005) The phylogeny and taxonomy of Hippopotamidae (Mammalia: Artiodactyla): a review based on morphology and cladistic analysis. Zoological Journal of the Linnean Society 143: 1-26.

84. Samonds KE (2007) Late Pleistocene bat fossils from Anjohibe Cave, northwestern Madagascar. Acta Chiropterologica 9: 39-65.

85. Russell AL, Goodman SM, Cox MP (2008) Coalescent analyses support multiple mainland-to-island dispersals in the evolution of Malagasy *Triaenops* bats (Chiroptera: Hipposideridae). Journal of Biogeography 35: 995-1003.

86. Horvath JE, Weisrock DW, Embry SL, Fiorentino I, Balhoff JP, et al. (2008) Development and application of a phylogenomic toolkit: resolving the evolutionary history of Madagascar's lemurs. Genome Research 18: 489-499.

87. Springer MS, Meredith RW, Gatesy J, Emerling CA, Park J, et al. (2012) Macroevolutionary dynamics and historical biogeography of primate diversification inferred from a species supermatrix. PLoS ONE 7: e49521.

88. Krause DW (2001) Fossil molar from a Madagascan marsupial. Nature 412: 497-498.

89. Jones KE, Bininda-Emonds ORP, Gittleman JL (2005) Bats, clocks, and rocks: Diversification patterns in Chiroptera. Evolution 59: 2243-2255.

90. Lamb JM, Ralph TMC, Naidoo T, Taylor PJ, Ratrimomanarivo F, et al. (2011) Toward a molecular phylogeny for the Molossidae (Chiroptera) of the Afro-Malagasy region. Acta Chiropterologica 13: 1-16.

91. Krause DW, O'Connor PM, Rogers KC, Sampson SD, Buckley GA, et al. Late Cretaceous terrestrial vertebrates from Madagascar: implications for Latin American biogeography; 2006. Annals of the Missouri Botanical Garden. pp. 178-208.

92. Samonds KE, Parent SN, Muldoon KM, Crowley E, Godfrey LR (2010) Rock matrix surrounding subfossil lemur skull yields diverse collection of mammalian subfossils: implications for reconstructing Madagascar’s paleoenvironments. Malagasy Nature 4: 1-16.

93. O'Brien J, Mariani C, Olson L, Russell AL, Say L, et al. (2009) Multiple colonisations of the western Indian Ocean by *Pteropus* fruit bats (Megachiroptera: Pteropodidae): the furthest islands were colonised first. Molecular Phylogenetics and Evolution 51: 294-303.

94. Goodman SM, Chan L, Nowak M, Yoder A (2010) Phylogeny and biogeography of western Indian Ocean *Rousettus* (Chiroptera: Pteropodidae). Journal of Mammalogy 91: 593-606.

95. Krause DW, Prasad GVR, von Koenigswald W, Sahni A, Grine FE (1997) Cosmopolitanism among Gondwanan Late Cretaceous mammals. Nature 390: 504-507.

96. Poux C, Madsen O, Glos J, de Jong WW, Vences M (2008) Molecular phylogeny and divergence times of Malagasy tenrecs: influence of data partitioning and taxon sampling on dating analyses. BMC Evolutionary Biology 8.

97. Stadelmann B, Jacobs D, Schoeman C, Ruedi M (2004) Phylogeny of African *Myotis* bats (Chiroptera, Vespertilionidae) inferred from cytochrome b sequences. Acta Chiropterologica 6: 177-192.

98. Trujillo RG, Patton JC, Schlitter DA, Bickham JW (2009) Molecular phylogenetics of the bat genus *Scotophilus* (Chiroptera: Vespertilionidae): Perspectives from paternally and maternally inherited genomes. Journal of Mammalogy 90: 548-560.

1. Scored as dispersal-advantaged due to saltwater-tolerant hard-shelled eggs, and demonstrated dispersal ability. [↑](#footnote-ref-1)
2. Scored as dispersal-advantaged as they are “semi-marine” and have demonstrated dispersal ability. [↑](#footnote-ref-2)
